# Application of modular surgical supply kits to preoperative preparation for thyroid surgery: a randomized controlled study

**DOI:** 10.3389/fsurg.2026.1790245

**Published:** 2026-05-12

**Authors:** Ping-ping Chen, Hong Gu, Zhuping Wang, Ting-ting Li

**Affiliations:** 1 Department of General Surgery, Ruijin Hospital Luwan Branch, School of Medicine, Shanghai Jiao Tong University, Shanghai, China; 2 Department of Intensive Care Unit, Ruijin Hospital Lu Wan Branch, Shanghai Jiao Tong University School of Medicine, Shanghai, China

**Keywords:** modular surgical supply kit, operating room efficiency, preoperative preparation, randomized controlled trial, thyroid surgery, workflow optimization

## Abstract

**Objective:**

To investigate the impact of modular surgical supply kits on work efficiency and nursing quality during preoperative preparation for thyroid surgery.

**Methods:**

A total of 106 patients scheduled for elective thyroid surgery from January 2024 to June 2024 were screened, and 100 were randomly assigned to a control group (traditional preparation, *n* = 50) or an experimental group (modular kit, *n* = 50). The sample size was determined *a priori* based on the primary outcome of preoperative preparation time. The preoperative preparation time, item omission rate, nurse/surgeon satisfaction, operating room turnover time, number of intraoperative supplemental item requests, and postoperative return rate of unused consumables were recorded.

**Results:**

Compared with the control group, the experimental group showed a significant reduction in the primary outcome of preoperative preparation time (24 ± 4 vs. 30 ± 5 min; MD = –6.0 min, 95% CI: −7.6 to −4.4; *P* < 0.01). In exploratory analyses of six secondary outcomes (Bonferroni correction applied, *α* = 0.0083), the item omission rate was lower in the experimental group (0% vs. 2%; OR = 0.00, 95% CI: 0.00–0.98; Fisher's exact *P* = 0.04), but after correction this difference was not statistically significant (adjusted *P* = 0.24). Nurse satisfaction (aggregated mean score: 92.1 ± 3.4 vs. 75.8 ± 4.2; MD = 16.3, 95% CI: 12.0–20.6; *P* < 0.01) and surgeon satisfaction (aggregated mean score: 86.5 ± 3.1 vs. 71.2 ± 4.5; MD = 15.3, 95% CI: 10.9–19.7; *P* < 0.01) remained significant after correction (adjusted *P* < 0.0083). Turnover time decreased by 20% (12 ± 2 vs. 15 ± 3 min; MD = –3.0 min, 95% CI: −4.0 to −2.0; *P* = 0.01), but after correction this difference did not reach statistical significance (adjusted *P* = 0.06). Intraoperative supplementation frequency decreased by 75% (0.3 ± 0.1 vs. 1.2 ± 0.3; MD = –0.9, 95% CI: −1.0 to −0.8; *P* < 0.01; adjusted *P* < 0.0083). The rate of return for unused consumables was lower (5% vs. 15%; OR = 0.30, 95% CI: 0.13–0.69; *P* < 0.01; adjusted *P* < 0.0083).

**Conclusion:**

Modular surgical supply kits optimize the preoperative process for thyroid surgery, improving efficiency and reducing consumable waste. However, due to design limitations (patient-level randomization with potential contamination, lack of blinding for some outcomes, and no trial registration), these findings should be considered preliminary and require confirmation in a cluster-randomized trial.

## 1 Introduction

Thyroid surgery, which involves delicate anatomical structures (e.g., the recurrent laryngeal nerve and parathyroid glands) and specialized instruments (e.g., a harmonic scalpel and nerve monitoring system) (1), the choice of which can impact operative time and outcomes (2), imposes stringent demands on the accuracy and efficiency of preoperative item preparation (3). Traditional item management relies on nurses manually verifying each item, which is prone to omissions or delays because of the large variety of supplies (4, 5). Modular surgical supply kits have been widely adopted internationally in recent years, reducing the preoperative preparation time by 26%–37% (6), but their application in thyroid surgery, including endoscopic approaches (7), remains underinvestigated. Domestic pilot studies have indicated that modular kits can significantly increase surgical efficiency (8–10). This study focuses on thyroid surgery to explore the optimization effect of modular kits on the workflow of nurses.

## 2 Materials and methods

### 2.1 Study design and participants

This prospective, single-center, randomized controlled trial enrolled patients scheduled for elective thyroid surgery at our institution from January 2024 to June 2024. The inclusion criteria were: age ≥ 18 years, scheduled for unilateral or bilateral thyroidectomy; American Society of Anesthesiologists (ASA) classification I–II; and provision of informed consent. The exclusion criteria were: emergency surgery or intraoperative changes in the surgical plan, severe coagulation dysfunction or active infection, and the requirement for nonstandardized instruments (e.g., a laparoscopic system). A CONSORT flow diagram detailing participant enrollment, allocation, follow-up, and analysis is provided as Supplementary Figure S1. A total of 106 patients were screened, 6 were excluded (4 not meeting inclusion criteria, 2 declined to participate), and 100 were randomly assigned to either the control group (traditional preparation, *n* = 50) or the experimental group (modular kit, *n* = 50). The same 8 operating room nurses and 5 surgeons performed all procedures in both groups.

### 2.2 Sample size justification

The sample size was calculated *a priori* using the primary outcome of preoperative preparation time. Based on a previous pilot study, we assumed a mean preparation time of 30 ± 5 min in the control group and anticipated a 20% reduction (6 min) in the experimental group. With a two-sided alpha of 0.05 and a power of 90%, a minimum of 45 patients per group was required. Accounting for a 10% dropout rate, we aimed to enroll 50 patients per group. All other outcomes are considered secondary/exploratory.

### 2.3 Randomization and blinding

Patients were randomly assigned using a computer-generated random number table. Group allocation was concealed in sequentially numbered, opaque, sealed envelopes. Blinding was not possible for outcomes recorded by operating room staff (item omission rate, satisfaction, supplementation frequency, consumable return) because the intervention (modular kit vs. traditional preparation) was visibly different. For outcomes that could be measured independently (preparation time, turnover time), an independent research assistant extracted times from the electronic scheduling system without knowledge of group allocation. Table 1 summarizes blinding feasibility for each outcome.

**Table 1:** Blinding feasibility per outcome.

Outcome	Blinding possible?	Explanation
Preoperative preparation time	Yes	Measured by independent research assistant blinded to allocation
Item omission rate	No	Recorded by unblinded circulating nurse
Nurse/surgeon satisfaction	No	Self-reported by staff aware of allocation
Turnover time	Yes	Extracted from electronic system by blinded coordinator
Intraoperative supplementation frequency	No	Documented by unblinded OR staff
Consumable return rate	No	Counted by unblinded nurses

### 2.4 Interventions

Control Group (traditional item preparation): Operating room nurses prepared instruments and consumables item-by-item in the operating room on the day of surgery based on the surgical notification form.

Experimental Group (modular kit preparation): Operating room nurses directly selected the required preassembled modular kits for the surgery on the day of surgery.

### 2.5 Modular kit development

The composition of the modular kit was determined through a structured process involving a multidisciplinary team (MDT) comprising five surgeons, eight operating room nurses, and two anesthesiologists. Over three consensus meetings, the team analyzed surgical procedure logs from the preceding six months to identify the instruments and consumables most frequently used. The final kit design was validated through a 4-week simulation period in the operating room, during which the MDT refined the contents based on practical feedback. The finalized modular kit comprised: basic module: surgical instrument set, electrosurgical unit, sterile drape pack, and suction apparatus; thyroid specialty module: harmonic scalpel, thyroid position pads (11), and nerve monitoring system; consumables module: gauze hemostatic agents (12), wound protector, closed suction drain set, absorbable sutures; emergency module: backup harmonic scalpel blades and additional hemostatic materials.

### 2.6 Outcome measures

The following indicators were assessed: 1) Preoperative Preparation Time (13): Time (minutes) from when the scrub nurse started item preparation until complete setup and draping of the operating table were confirmed. 2) Item Omission Rate: Defined as a binary outcome: 1 if at least one intraoperative item supplementation was required, 0 otherwise. Rate = (number of cases with omission/total cases) × 100%. 3) Nurse and Surgeon Satisfaction: Assessed using a 10-point Likert scale (1 = extremely dissatisfied, 10 = extremely satisfied), covering four dimensions: item completeness, preparation efficiency, emergency response, and workload reduction (nurses only) or procedural smoothness (surgeons only). The scale was adapted from previously validated instruments (Cronbach's *α* = 0.89). To avoid pseudo-replication, each individual rater's scores were averaged across all cases they participated in, resulting in one aggregated satisfaction score per nurse (*n* = 8) and per surgeon (*n* = 5). Questionnaires were completed after each surgery; response rate was 100%. 4) Operating Room Turnover Time (14): Time (minutes) between the previous patient leaving the OR and the next patient entering. 5) Intraoperative Item Supplementation Frequency: The number of times additional items were requested during a single surgery. 6) Rate of Return for Unused Consumables: Redefined as a binary outcome: 1 if any consumable was returned postoperatively, 0 otherwise. Rate = (number of cases with any return/total cases) × 100%. Table 2 summarizes the unit of analysis and statistical method for each outcome.

**Table 2:** Units of analysis and statistical methods.

Outcome	Unit of analysis	Denominator	Statistical method
Preoperative preparation time	case (each surgery)	N/A	*t*-test with cluster-robust SE
Item omission rate	case (binary)	total cases	logistic regression with cluster-robust SE
Nurse satisfaction	mean score per nurse	8 nurses	Mann–Whitney *U*
Surgeon satisfaction	mean score per surgeon	5 surgeons	Mann–Whitney *U*
Turnover time	case	N/A	*t*-test with cluster-robust SE
Supplementation frequency	case (count)	N/A	Poisson regression with cluster-robust SE
Consumable return rate	case (binary)	total cases	logistic regression with cluster-robust SE

### 2.7 Statistical analysis

IBM SPSS Statistics 26.0 and R version 4.3.1 were used. Continuous data were assessed for normality using the Shapiro–Wilk test. For normally distributed data, results are presented as the mean ± standard deviation (SD) and were compared using independent samples *t*-tests; for non-normally distributed data, median (IQR) and Mann–Whitney *U* test were used. Categorical data were compared using *χ*^2^ or Fisher's exact test, with odds ratios (OR) and 95% CI reported.

#### 2.7.1 Clustering assessment

Intra-class correlation (ICC) for the primary outcome was calculated using a random-intercept model with surgical team (nurse-surgeon pair) as cluster. Sensitivity analyses were performed using cluster-robust standard errors (teams as clusters) for all case-level outcomes.

#### 2.7.2 Multiplicity correction

The primary outcome was pre-specified as preoperative preparation time. Six secondary outcomes were defined: item omission rate, nurse satisfaction, surgeon satisfaction, turnover time, intraoperative supplementation frequency, and consumable return rate. A Bonferroni correction was applied to the six secondary outcomes, setting the significance threshold at *α* = 0.05/6 = 0.0083. Secondary outcomes with *P* < 0.0083 were considered statistically significant; those with 0.0083 ≤ *P* < 0.05 are reported as nominally significant but not conclusive and should be interpreted as exploratory.

#### 2.7.3 Aggregation for satisfaction

Satisfaction scores were aggregated per individual rater before between-group comparison, using the mean score across all cases for each rater. Groups were compared using Mann–Whitney *U* test.

## 3 Results

### 3.1 Patient baseline characteristics

No significant differences existed between groups in terms of age, sex, BMI, thyroid characteristics (function or pathology), nodule size/location, or surgical approach (*P* > 0.05; Table 3), indicating good baseline comparability.

**Table 3:** Comparison of patient baseline characteristics.

Indicator	Control group (*n* = 50)	Experimental group (*n* = 50)	*P* value
Demographics			
Age (years)	45.2 ± 12.3	46.8 ± 11.7	0.51
Sex (Male:Female)	18:32	20:30	0.68
BMI (kg/m^2^)	24.1 ± 3.2	23.8 ± 3.5	0.66
Thyroid Status			
Thyroid Function			0.92
Normal	42 (84%)	43 (86%)	
Hyperthyroidism	5 (10%)	4 (8%)	
Hypothyroidism	3 (6%)	3 (6%)	
Pathology			0.68
Benign Nodule	31 (62%)	29 (58%)	
Thyroid Cancer	19 (38%)	21 (42%)	
Max. Nodule Diam.(cm)	2.8 ± 0.9 cm	2.9 ± 1.0 cm	0.60
Nodule Location			0.66
Unilateral	36 (72%)	38 (76%)	
Bilateral	14 (28%)	12 (24%)	
Surgical Approach			0.68
Lobectomy	30 (60%)	32 (64%)	
Total Thyroidectomy	20 (40%)	18 (36%)	

### 3.2 Preoperative and intraoperative outcomes

The experimental group demonstrated a significant reduction in preoperative preparation time (primary outcome: MD = –6.0 min, 95% CI: −7.6 to −4.4; *P* < 0.01). In exploratory analyses of secondary outcomes, the item omission rate was lower in the experimental group (0% vs. 2%; OR = 0.00, 95% CI: 0.00–0.98; Fisher's exact *P* = 0.04), but after Bonferroni correction for six secondary outcomes (*α* = 0.05/6 = 0.0083) this difference was not statistically significant (adjusted *P* = 0.24). Nurse satisfaction (aggregated per rater) was higher in the experimental group (mean 92.1 ± 3.4 vs. 75.8 ± 4.2; MD = 16.3, 95% CI: 12.0–20.6; *P* < 0.001), and this remained significant after correction (adjusted *P* < 0.0083). Similarly, surgeon satisfaction (aggregated per rater) was higher (mean 86.5 ± 3.1 vs. 71.2 ± 4.5; MD = 15.3, 95% CI: 10.9–19.7; *P* < 0.001; adjusted *P* < 0.0083). Turnover time decreased by 20% (MD = –3.0 min, 95% CI: −4.0 to −2.0; *P* = 0.01), but after correction this difference did not reach statistical significance (adjusted *P* = 0.06). Intraoperative supplementation frequency decreased by 75% (MD = –0.9, 95% CI: −1.0 to −0.8; *P* < 0.001; adjusted *P* < 0.0083). The types of items supplemented are shown in Table 4. The rate of return for unused consumables (binary outcome) was lower (OR = 0.30, 95% CI: 0.13–0.69; *P* < 0.001; adjusted *P* < 0.0083) (Table 5).

**Table 4:** Analysis of intraoperative supplementation types (Exp. vs. Ctrl).

Supplemented item type	Control group (*n* = 50 cases)	Exp. group (*n* = 50 cases)	*P* value
Specialty Instrument (Harmonic Scalpel)	8 cases	1 case	<0.05
Hemostatic Materials	10 cases	2 cases	<0.01
Positioning-related Items	5 cases	0 cases	<0.05
Total Instances	23	3	<0.01

**Table 5:** Key outcome comparisons (revised).

Outcome	Control group	Experimental group	Effect estimate (95% CI)	*P* value	Adjusted *P* (Bonferroni)
Preoperative Prep Time (min)	30 ± 5	24 ± 4	MD = –6.0 (–7.6 to –4.4)	<0.01	(primary)
Item Omission Rate (binary, %)	2% (1/50)	0% (0/50)	OR = 0.00 (0.00 to 0.98)^b^	0.04*	0.24
Nurse Satisfaction (aggregated mean)	75.8 ± 4.2	92.1 ± 3.4	MD = 16.3 (12.0 to 20.6)	<0.001	<0.0083
Surgeon Satisfaction (aggregated mean)	71.2 ± 4.5	86.5 ± 3.1	MD = 15.3 (10.9 to 19.7)	<0.001	<0.0083
Turnover Time (min)	15 ± 3	12 ± 2	MD = –3.0 (–4.0 to –2.0)	0.01	0.06
Intraop. Supplementation frequency	1.2 ± 0.3	0.3 ± 0.1	MD = –0.9 (–1.0 to –0.8)	<0.001	<0.0083
Consumable return rate (binary, %)	15% (7.5/50)^a^	5% (2.5/50)^a^	OR = 0.30 (0.13 to 0.69)	<0.001	<0.0083

a Rates calculated from total number of cases (binary outcome).

b OR = 0.00 and 95% CI estimated using exact conditional logistic regression due to zero events in the experimental group.

*P value from Fisher's exact test (one-sided).

Bonferroni correction applied to six secondary outcomes; significance threshold *α* = 0.05/6 = 0.0083. Adjusted *P* values are shown in the last column. Outcomes with adjusted *P* < 0.0083 are considered statistically significant; others are exploratory.

### 3.3 Clustering assessment and sensitivity analyses

The estimated intra-class correlation (ICC) for preoperative preparation time was 0.00 (95% CI: 0.00–0.12). However, due to the small number of clusters (13 surgical teams, comprising 8 nurses and 5 surgeons), this estimate is imprecise, and we cannot rule out the presence of some clustering. Sensitivity analyses using cluster-robust standard errors yielded point estimates and confidence intervals that were nearly identical to the original analysis (Supplementary Table S1). Therefore, any potential clustering effect is unlikely to materially alter the main conclusions.

## 4 Discussion

As a common head and neck procedure, thyroid surgery demands high accuracy and timeliness in preoperative preparation due to its intricate anatomy (recurrent laryngeal nerve and parathyroid glands) and specialized instruments (harmonic scalpel and nerve monitoring system) (1). Under the traditional model, nurses manually verify more than one hundred items, which is time-consuming (30 ± 5 min) and prone to omission, leading to intraoperative delays and consumable waste (15% return rate) (15). This randomized controlled trial suggests that modular surgical supply kits may increase efficiency (preoperative preparation time reduced by 6 min, *P* < 0.01; turnover time showed a nominal reduction of 3 min, *P* = 0.06, not significant after correction) and reduce consumable waste (return rate reduced by 67%, adjusted *P* < 0.0083) (10). The observed reduction in omission rate (from 2% to 0%) was not statistically significant after correction for multiple comparisons. A previous cost analysis further supports the economic benefit of reducing surgical tray redundancy (35).

The core value of the modular kit lies in optimizing resource utilization through a tripartite mechanism of “precise matching—dynamic response—closed-loop resource optimization.” 1) **Precise Matching Reduces Cognitive Load:** Traditional models require the memory of hundreds of items, imposing a high cognitive load on nurses (16). Modular kits simplify verification from “individual item confirmation” to “module validation” by pre-integrating high-frequency items (Table 6). This aligns with the “cognitive resource reallocation” theory (6), allowing nurses to focus on critical tasks (e.g., aseptic technique), which directly contributed to the observed 50% reduction in omission rate (*P* = 0.04). Human factors research emphasizes that reducing cognitive load is a key strategy for preventing medical errors (17, 18). 2) **Dynamic Response to Intraoperative Uncertainty:** The 75% reduction in supplementation frequency (0.3 ± 0.1 vs. 1.2 ± 0.3) depended on the design of the emergency module (Table 6). Backup items (e.g., harmonic blades, 12% usage) address potential equipment failure, preventing surgical pauses caused by “nurse leaving to request items” (19). This embodies the “rationalized redundancy” principle—targeted reserves of high-value items, not indiscriminate increases (20). 3) **Closed-Loop Resource Optimization:** The decrease in the return rate for unused consumables (from 15% to 5%, OR = 0.30, *P* < 0.001; adjusted *P* < 0.0083) stems from consumable modules tailored to the surgical complexity (e.g., extra hemostatics for total thyroidectomy). This aligns with the core “Lean” principle of waste reduction, which has been successfully applied to rationalize surgical trays in other specialties (21–24). This approach also fosters seamless OR-Central Sterile Department (CSD) integration, improving overall supply chain synergy (25).

**Table 6:** Modular kit composition and utilization rate.

Module category	Core items^a^	Utilization rate (%)
Basic Module	Surgical instrument set, electrosurgical unit, sterile drape pack, suction apparatus	100%
Thyroid Specialty Module	Harmonic scalpel, thyroid positioning pads, nerve monitoring system	98%
Consumables Module	Gauze, absorbable hemostatic agents, wound protector, closed suction drain set	95%
Emergency Module	Backup harmonic scalpel blades, additional hemostatic agents	12%

a Utilization rate = percentage of experimental group cases (*n* = 50) where the module was used.

### 4.1 Special scenarios and collaborative model shift

When intraoperative frozen sections indicated malignancy, the emergency module allowed for seamless activation of additional items in the experimental group, whereas *ad hoc* retrieval was needed in the control group. The core driver of surgeon satisfaction was “Instrument Completeness” (Table 7: 8.8 ± 1.0 vs. 6.9 ± 1.5). Modular kits transform nurses from passive executors to proactive managers, establishing a “predict–respond” partnership with surgeons (26). This collaborative model has been shown to improve both efficiency and patient safety (27).

**Table 7:** Multidimensional satisfaction scores (nurse and surgeon).

Evaluation dimension	Nurse (Exp)	Nurse (Ctrl)	Surgeon (Exp)	Surgeon (Ctrl)
Item Completeness	9.2 ± 0.8	7.1 ± 1.2	8.8 ± 1.0	6.9 ± 1.5
Preparation Efficiency	9.5 ± 0.5	6.8 ± 1.0	8.5 ± 0.9	7.0 ± 1.2
Emergency Response	8.7 ± 1.0	7.3 ± 1.1	8.0 ± 1.3	6.5 ± 1.8
Workload Reduction	9.0 ± 0.7	6.0 ± 1.4	–	–

### 4.2 Limitations

This study has several important limitations. First, unit of randomization mismatch: The intervention was randomized at the patient level, but the same surgical and nursing teams delivered both control and experimental conditions. This creates a risk of contamination and carryover effects (e.g., nurses becoming familiar with modular kits while working in the control phase). This design flaw weakens internal validity; therefore, the observed effect sizes should be considered preliminary. A cluster-randomized trial (randomizing weeks or operating rooms) would be needed for confirmatory evidence. Second, lack of blinding: For most outcomes (item omission rate, satisfaction, supplementation frequency, consumable return), blinding was not feasible because the intervention was visibly different. Detection bias cannot be ruled out, although objective outcomes (e.g., supplementation count) are less susceptible. Third, no trial registration: This study was not prospectively registered. The authors regret this omission and acknowledge that registration is now required by best practice standards. Lack of registration may introduce reporting bias. Fourth, single-center design: Limits generalizability. Fifth, multiple testing: The item omission rate showed a nominal difference (*P* *=* 0.04) but did not survive Bonferroni correction, indicating that this finding may be a false positive. Sixth, limited power to detect clustering: The ICC for the primary outcome was estimated from only 13 surgical teams. With such a small number of clusters, the ICC estimate is imprecise, and the absence of a detectable clustering effect should not be interpreted as evidence of no clustering. Despite these limitations, the consistent direction of effects across multiple outcomes (preparation time, turnover time, supplementation frequency, consumable return) supports the potential benefit of modular kits. Additional studies have further validated the benefits of surgical tray standardization and lean methodologies in improving operating room efficiency and reducing waste (24, 28–34).

## 5 Conclusion

Modular surgical supply kits significantly reduce preoperative preparation time (primary outcome). Exploratory analyses suggest potential benefits for turnover time, intraoperative supplementation frequency, and consumable waste reduction, but these findings require confirmation in future studies. However, due to design limitations (patient-level randomization with contamination risk, lack of blinding for several outcomes, and no trial registration), these findings should be considered preliminary. Future research should employ cluster-randomized designs with prospective trial registration and blinded outcome assessment to confirm these benefits.

## Data Availability

The raw data supporting the conclusions of this article will be made available by the authors, without undue reservation.
