# Supplementary material for: Application of modular surgical supply kits to preoperative preparation for thyroid surgery: a randomized controlled study
Source: Front Surg. 2026 May 12;13:1790245. doi: 10.3389/fsurg.2026.1790245 (PMC13201439; doi:10.3389/fsurg.2026.1790245)
Supplement: Supplementary file 1 [file Datasheet1.pdf]

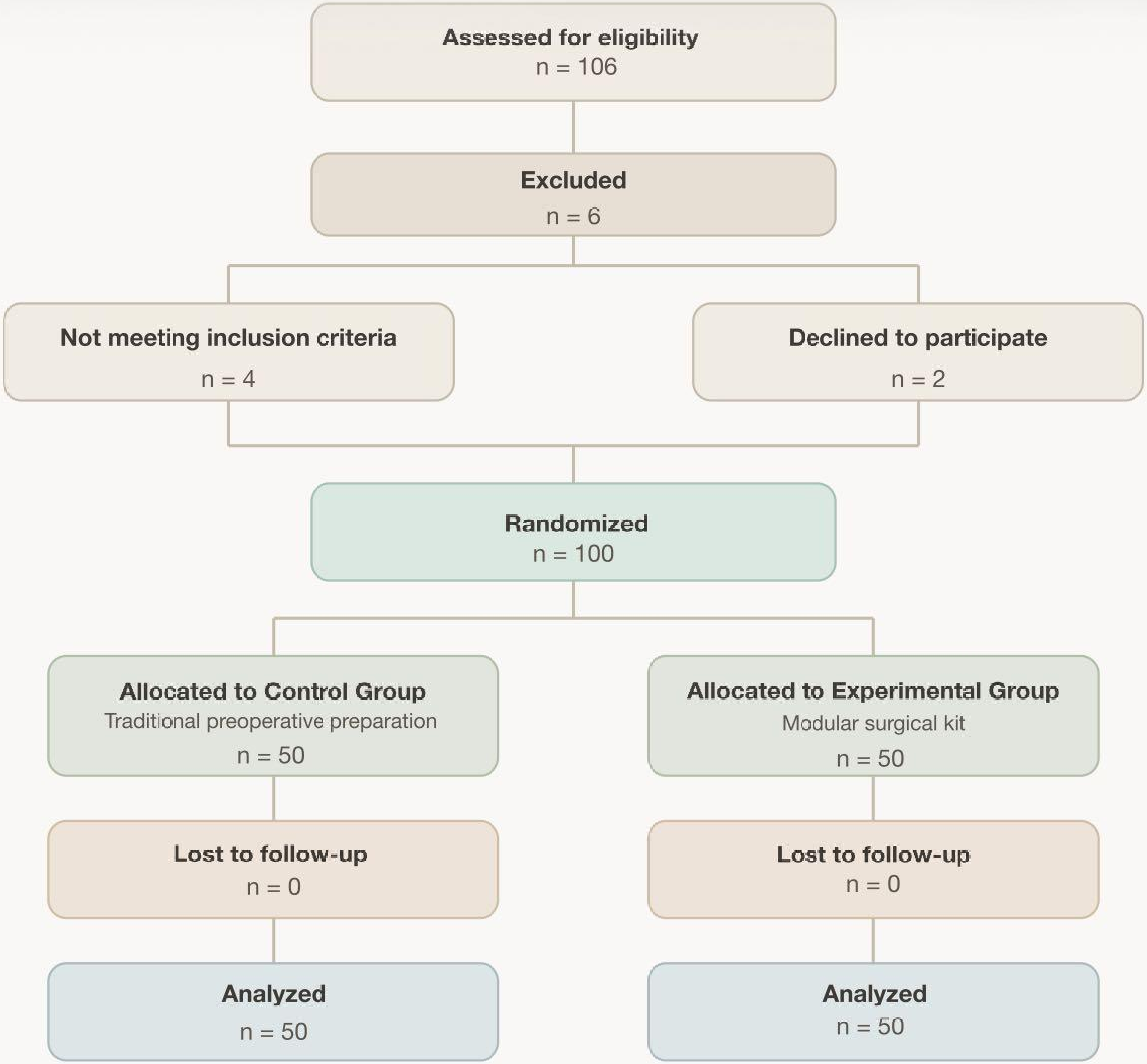

Supplementary Figure 1.
CONSORT flow diagram of the study. A total of 106 patients were assessed for eligibility; 6 were excluded (4 not meeting inclusion criteria, 2 declined to participate); 100 were randomized (50 to control group, 50 to experimental group); no patients were lost to follow-up; all 100 were included in the final analysis.
