# Supplementary material for: Application of modular surgical supply kits to preoperative preparation for thyroid surgery: a randomized controlled study
Source: Front Surg. 2026 May 12;13:1790245. doi: 10.3389/fsurg.2026.1790245 (PMC13201439; doi:10.3389/fsurg.2026.1790245)
Supplement: Supplementary file 2 [file Datasheet2.pdf]

Supplementary Table S1.
Sensitivity analysis using cluster-robust standard errors

Outcome	Original estimate (95% CI)	Cluster-robust estimate (95% CI)	ICC	Change
Preoperative preparation time (MD, min)	–6.0 (–7.6 to –4.4)	–6.0 (–7.6 to –4.4)	0.00	None
Item omission rate (OR)	0.00 (0.00 to 0.98)*	0.00 (0.00 to 0.98)*	–	None
Turnover time (MD, min)	–3.0 (–4.0 to –2.0)	–3.0 (–4.1 to –1.9)	–	Negligible
Intraoperative supplementation frequency (MD)	–0.9 (–1.0 to –0.8)	–0.9 (–1.0 to –0.8)	–	None
Consumable return rate (OR)	0.30 (0.13 to 0.69)	0.30 (0.12 to 0.72)	–	Slightly wider

Notes: MD = mean difference; OR = odds ratio; CI = confidence interval.

Cluster-robust standard errors were computed using the vcovCL function in R (package sandwich) with surgical team (nurse-surgeon pair, n = 13 teams) as clustering variable. ICC for primary outcome estimated from linear mixed-effects model with team as random intercept.

*For the item omission rate, the original analysis used Fisher's exact test; cluster-robust estimation gave an identical OR (0.00) and upper confidence limit (0.98). The lower bound is 0.00 due to zero events in the experimental group.
